# Supplementary material for: Human sleep spindles track experimentally excited brain circuits
Source: Sleep. 2025 Apr 28;48(7):zsaf114. doi: 10.1093/sleep/zsaf114 (PMC12246375; doi:10.1093/sleep/zsaf114)
Supplement: zsaf114_suppl_Supplementary_Figures_S1-S8_Tables_S1-S2 [file zsaf114_suppl_supplementary_figures_s1-s8_tables_s1-s2.docx]

***Supplementary material for***

**Human sleep spindles track experimentally excited brain circuits**

Jude L. Thom^1,2^, Bernhard P. Staresina^1,2,3^

1. *Department of Experimental Psychology, University of Oxford, Oxford, UK*
2. *Oxford Centre for Human Brain Activity, Wellcome Centre for Integrative Neuroimaging, Department of Psychiatry, University of Oxford, Oxford, UK*
3. *Corresponding Author*

Corresponding Author information:

Prof Bernhard Staresina

Address:

Department of Experimental Psychology,

Anna Watts Building, Woodstock Rd, Oxford,

OX2 6GG, United Kingdom

E-Mail:

bernhard.staresina@psy.ox.ac.uk


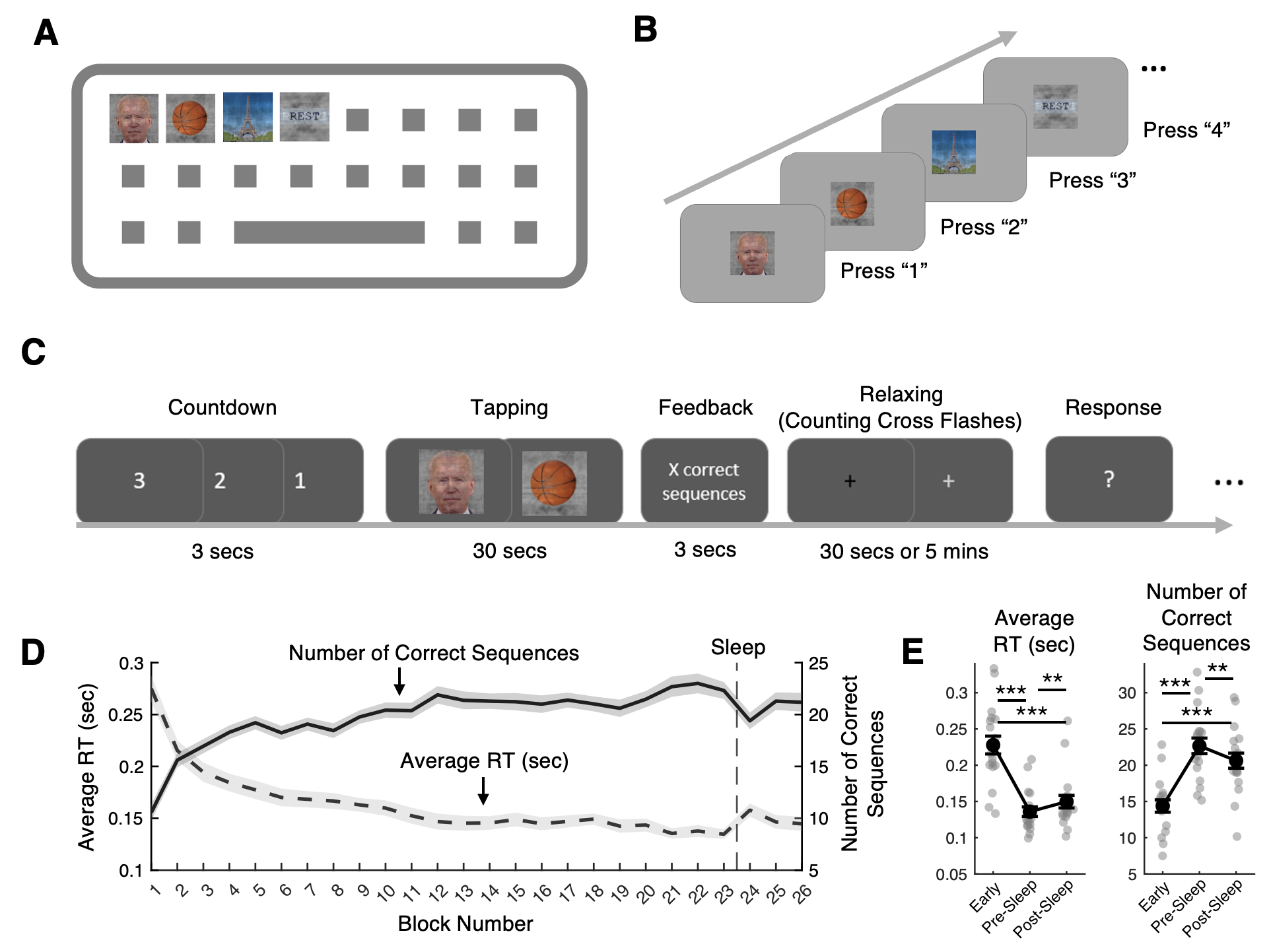


**Supplementary Figure 1.** *Overview of visuomotor finger-tapping task.* ***A)*** *An image category was assigned to each of the four number keys in the top row of the keyboard. The participant received explicit instructions to use one finger per key. The assignment was always 1-Face, 2-Object, 3-Scene, 4-Word, although the images were unique to each session and were counterbalanced across subjects.* ***B)*** *Pressing the image category key would progress the task to the next image.* ***C)*** *Overview of a block. After a countdown, the participant tapped for 30 secs before receiving feedback on the number of correct four-part sequences they achieved. They then relaxed their hand and counted the number of times the fixation cross changed brightness for 30 secs (5 mins after block 10 and block 20) and finally responded how many flashes they counted with their right hand on a separate keyboard. There were 23 blocks in total during the learning task and three blocks after sleep.* ***D)*** *Learning over blocks. Mean number of correct sequences (solid line) and mean average RT (dashed line) over blocks. Sessions were treated as independent (N = 38). Shading indicates ±1 SEM.* ***E)*** *Comparison of mean values of average RT and number of correct sequences across early blocks (blocks 1-3), pre-sleep blocks (blocks 21-23), and post-sleep blocks (blocks 24-26). The values were averaged across sessions for each subject before comparing early, pre-sleep, and post-sleep performance. Paired-sample t-tests showed significant decreases in average RT and increases in number of correct sequences from early (average RT: M = 0.23, SD = 0.05; number of correct sequences: M = 14.37, SD = 3.70) to pre-sleep (average RT: M = 0.14, SD = 0.03; number of correct sequences: M = 22.67, SD = 4.68) blocks (average RT: t(18) = 10.09, p < .001, d = 2.32, two-tailed; number of correct sequences: t(18) = -9.72, p < .001, d = -2.23, two-tailed). Performance became worse in post-sleep blocks (average RT: M = 0.15, SD = 0.04; number of correct sequences: M = 20.62, SD = 4.53), compared to pre-sleep blocks (average RT: t(18) = -3.36, p = .004, d = -0.77, two-tailed, paired-sample; number of correct sequences: t(18) = 3.87, p = .001, d = 0.89, two-tailed, paired-sample), while post-sleep performance remained significantly better than performance during early blocks (average RT: t(18) = 9.87, p < .001, d = 2.27, two-tailed, paired-sample; number of correct sequences: t(18) = -9.20, p < .001, d = -2.11, two-tailed, paired-sample).*


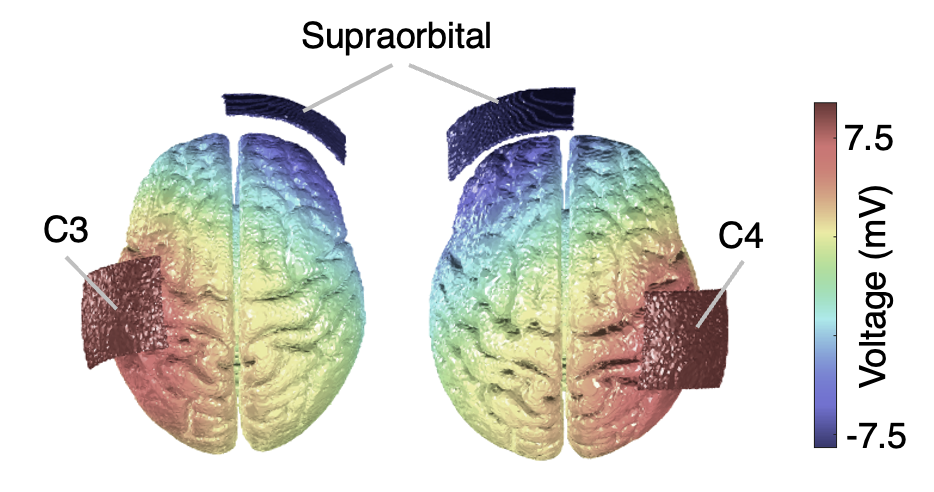


**Supplementary Figure 2.** *Simulation of tDCS voltage effects.* ***A)*** *Simulation using ROAST* ^84^ *of the distribution of voltage caused by 1mA tDCS stimulation for 20 mins during left excitatory and right excitatory stimulation. The simulation was conducted using a template brain.*

*
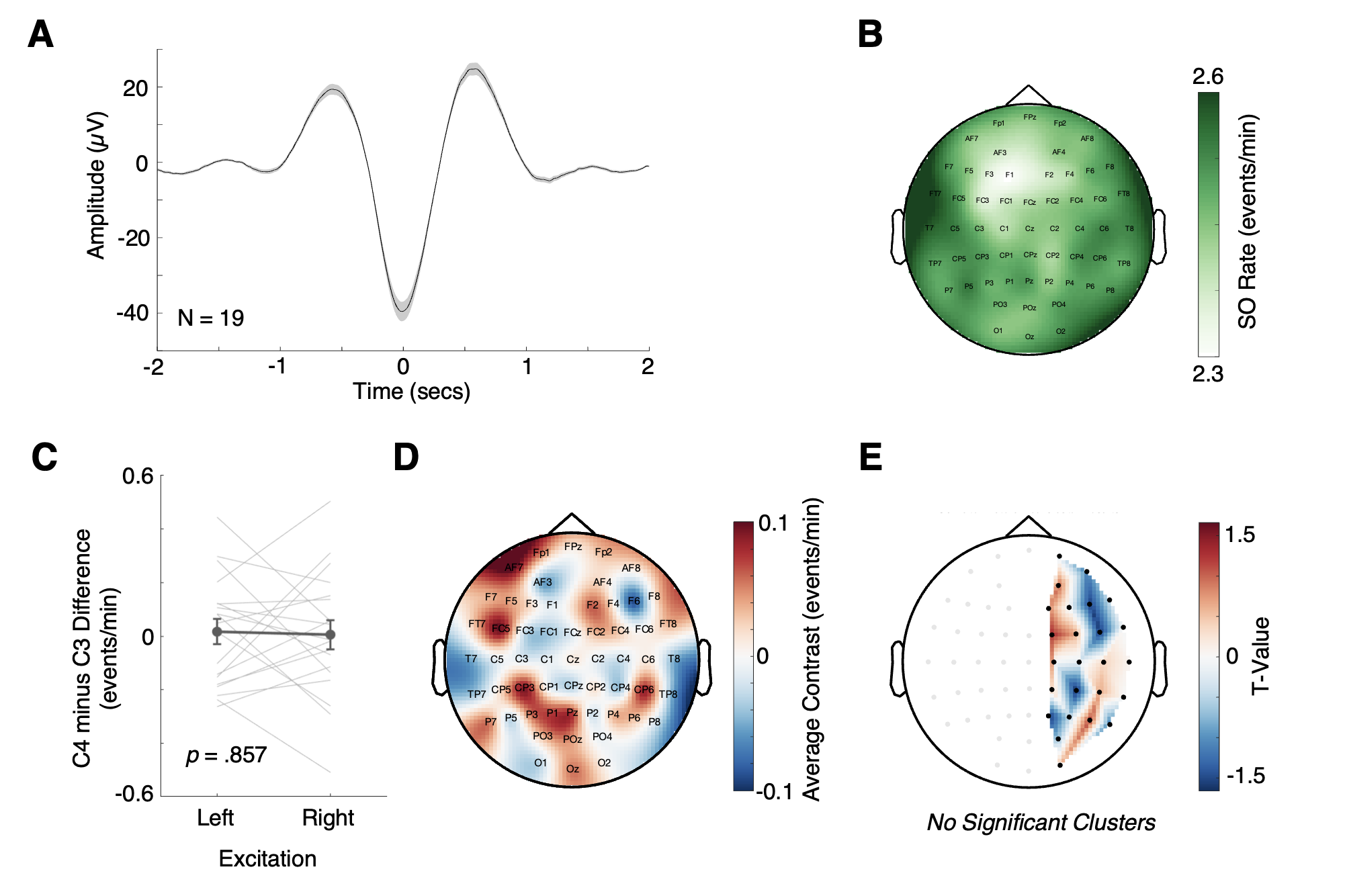
*

**Supplementary Figure 3.** *No evidence of tDCS stimulation influencing the lateralisation of SO rates.* ***A-E)*** *Same as* ***Figure 2B-C*** *and* ***Figure 3A-C*** *with SO rates. Lines represent individual participants. Error bars represent ±1 SEM. T-test was paired-sample and two-sided. Cluster-based permutation tests were tested as p<.05 significance.*

**
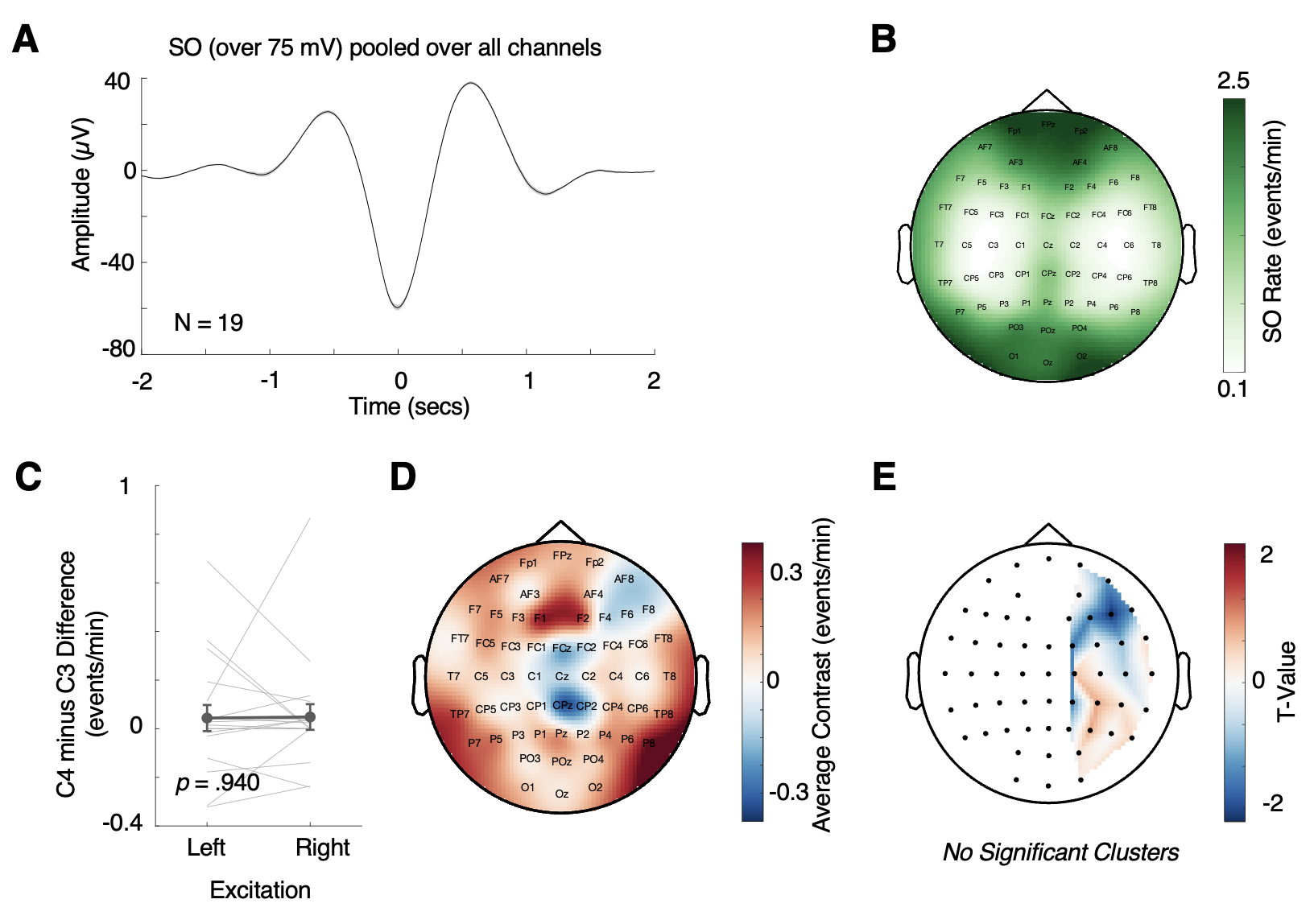
**

**Supplementary Figure 4.** *No evidence of tDCS stimulation influencing the lateralisation of SO rates, using >75 µV threshold.* ***A)*** *Grand average SO pooled across all contacts. SOs were aligned to their largest trough and baseline corrected using the mean of the 2 sec window, before averaging across sessions (N = 2) and across participants (N = 19). Shading represents ±1 SEM.* ***B-E)*** *Same as* ***Figure 3B-E****. Lines represent individual participants. Error bars represent ±1 SEM. T-test was paired-sample and two-sided. Cluster-based permutation tests were tested as p<.05 significance.*


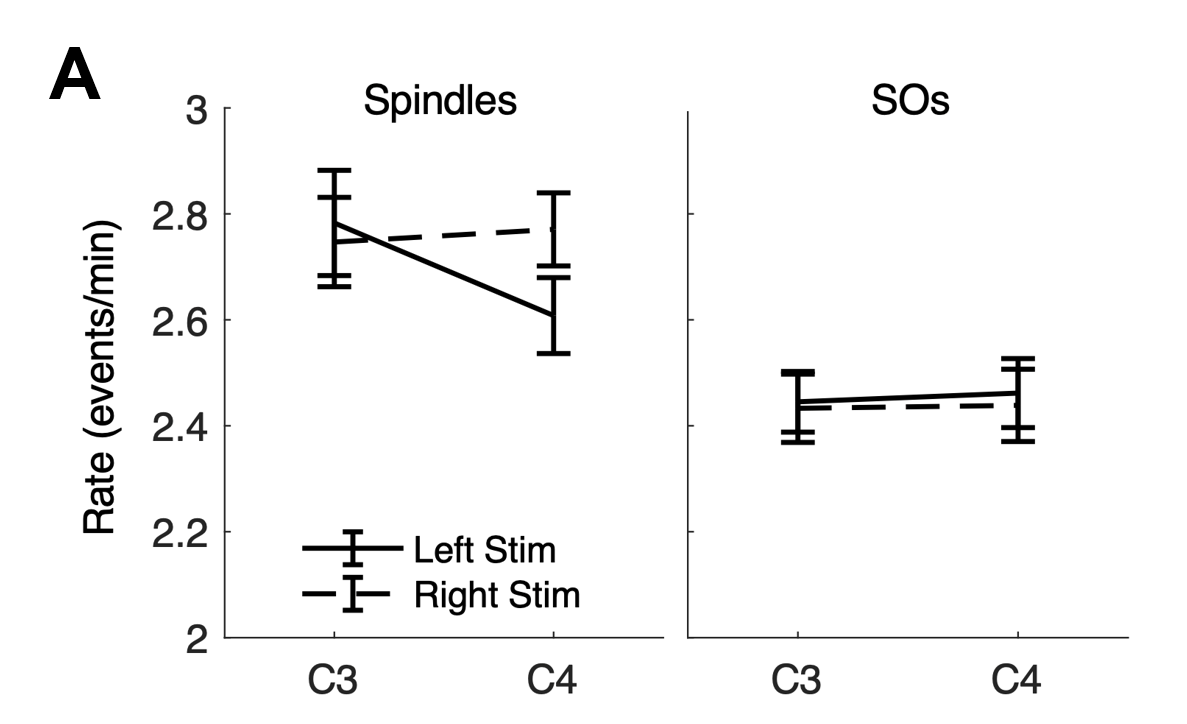


**Supplementary Figure 5.** *3-way ANOVA and interaction between event type, stimulation condition, and contact.* ***A)*** *The mean event rates across subjects, split by event type, contact, and stimulation condition. Error bars indicate ±1 SEM. The main effect of event type was significant, F(1,18) = 12.13, p = .003, η_p_^2^ = .40, with spindles having a greater rate than SOs (spindles, M = 2.73, SD = 0.36; SOs, M = 2.44, SD = 0.27). The 3-way interaction between event type, stimulation condition, and contact was significant (F(1,18) = 5.59, p = .030, η_p_^2^ = .24).*


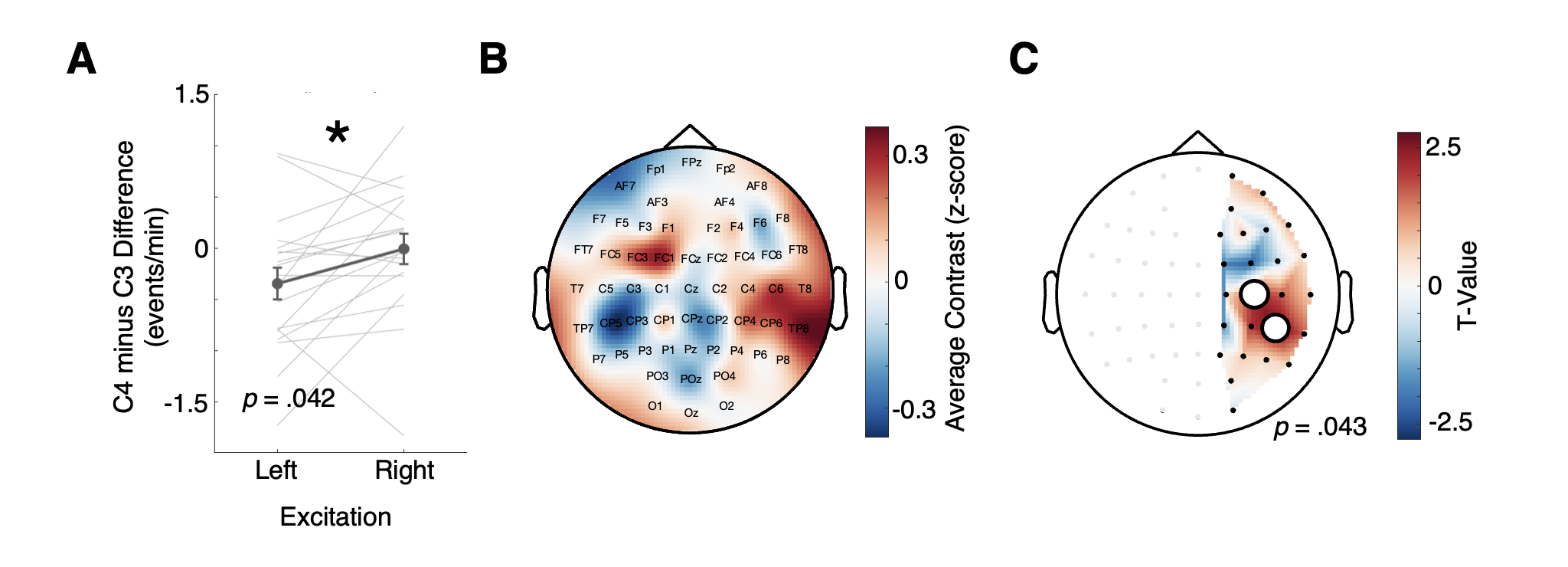


**Supplementary Figure 6.** *Z-scored* *spindle rates were influenced by lateralised tDCS stimulation.* ***A-C)*** *Same as* ***Figure 3*** *with z-scored spindle rates (normalised within each session before subtracting and creating contrasts). Error bars represent ±1 SEM. Lines represent individual participants. White circles highlight significant clusters, p < .05.*

*
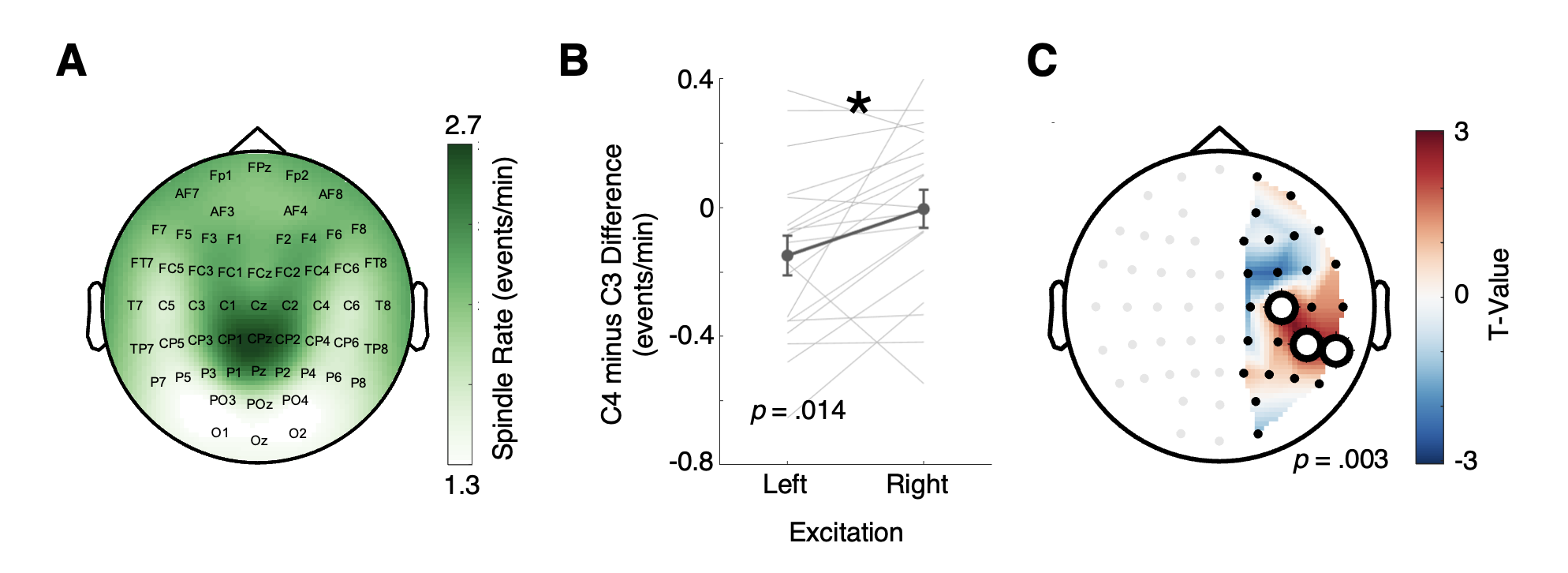
*

**Supplementary Figure 7.** *Spindle rates with more conservative thresholds were influenced by lateralised tDCS stimulation.* ***A-C)*** *Same as* ***Figures 2C, 3A, & 3C*** *with spindle rates using a detection threshold of mean + 1.75 SD (rather than mean + 1.5 SD). Lines represent individual participants. Error bars represent ±1 SEM. White circles highlight significant clusters, p < .05.*


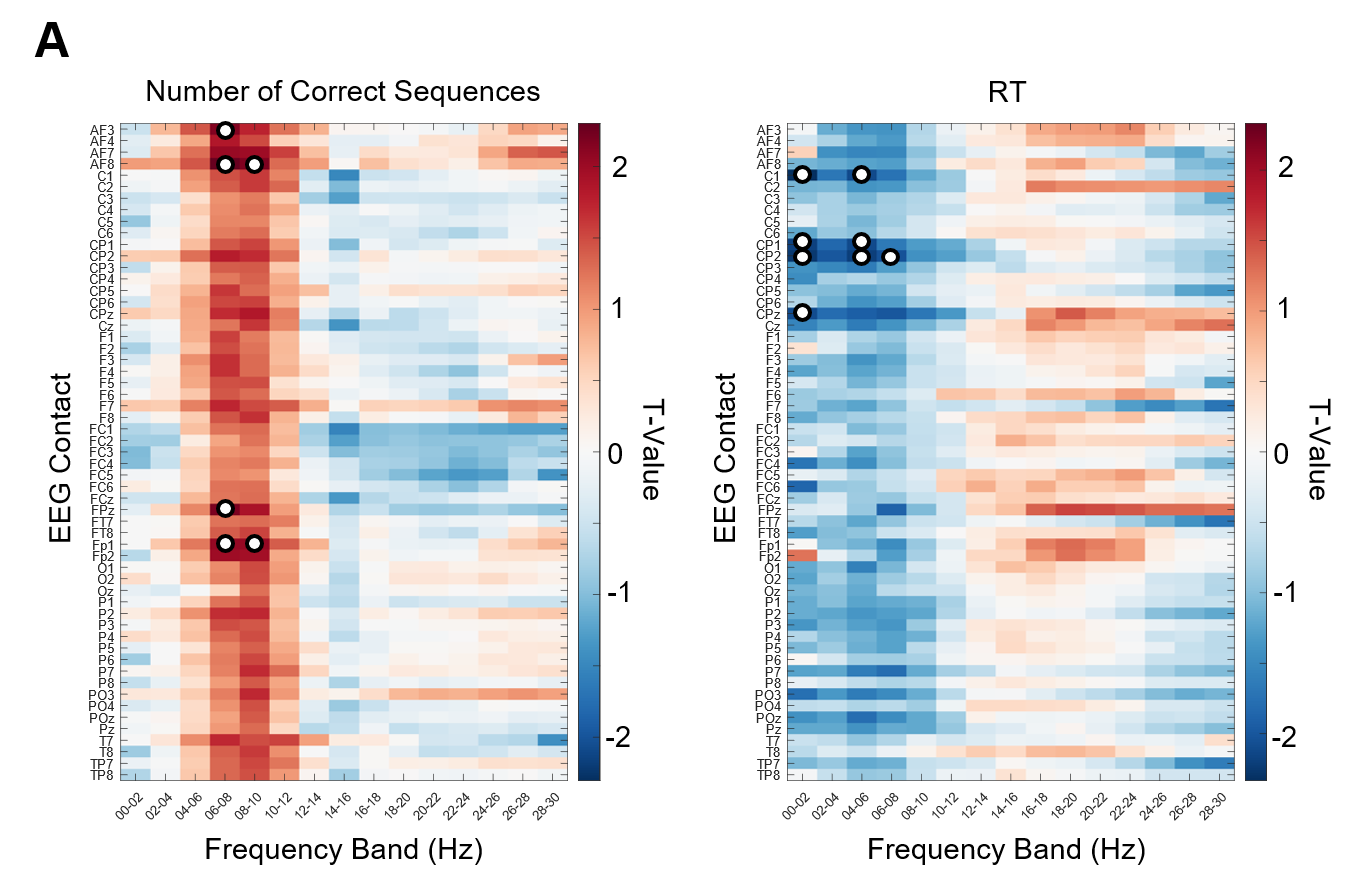


**Supplementary Figure 8.** *Exploration of relationship between spectral power in N2 / N3 sleep and visuomotor skill retention after sleep.* ***A)*** *The t-values for the effect of spectral power on skill retention from exploratory LME models with the equation ‘Retention ~ Session + Stimulation Site + Spectral Power + (1|Participant Number)’. One LME model was employed for each EEG contact-frequency bin combination (see* ***Methods*** *for details). Positive and negative t-values indicate better retention after sleep for the number of correct sequences and RT, respectively. White circles represent significance (p < .05), not corrected for multiple comparisons.*

| **Session** | **Metric** | **Mean** | **SD** | **Min** | **Max** |
| --- | --- | --- | --- | --- | --- |
| Both | Time N1 (mins) | 12.41 | 8.70 | 2.50 | 44.00 |
|  | Time N2 (mins) | 41.62 | 12.95 | 18.00 | 63.50 |
|  | Time N3 (mins) | 19.07 | 12.32 | 0.00 | 45.50 |
|  | Time N2/3 (mins) | 60.68 | 16.69 | 23.00 | 91.50 |
|  | Time REM (mins) | 5.64 | 8.16 | 0.00 | 29.00 |
|  | Total Sleep Time (mins) | 78.74 | 12.13 | 49.50 | 100.00 |
|  | Proportion N1 | 0.17 | 0.13 | 0.03 | 0.66 |
|  | Proportion N2 | 0.53 | 0.14 | 0.26 | 0.78 |
|  | Proportion N3 | 0.24 | 0.15 | 0.00 | 0.55 |
|  | Proportion N2/3 | 0.76 | 0.15 | 0.34 | 0.97 |
|  | Proportion REM | 0.07 | 0.10 | 0.00 | 0.35 |
| 1 | Time N1 (mins) | 12.42 | 7.83 | 3.50 | 37.00 |
|  | Time N2 (mins) | 41.97 | 9.85 | 18.00 | 59.00 |
|  | Time N3 (mins) | 19.89 | 12.42 | 0.00 | 45.50 |
|  | Time N2/3 (mins) | 61.87 | 15.65 | 26.00 | 87.50 |
|  | Time REM (mins) | 5.47 | 6.10 | 0.00 | 19.50 |
|  | Total Sleep Time (mins) | 79.76 | 12.27 | 50.50 | 100.00 |
|  | Proportion N1 | 0.17 | 0.12 | 0.04 | 0.52 |
|  | Proportion N2 | 0.53 | 0.13 | 0.26 | 0.69 |
|  | Proportion N3 | 0.24 | 0.14 | 0.00 | 0.53 |
|  | Proportion N2/3 | 0.77 | 0.13 | 0.37 | 0.93 |
|  | Proportion REM | 0.07 | 0.07 | 0.00 | 0.21 |
| 2 | Time N1 (mins) | 12.39 | 9.71 | 2.50 | 44.00 |
|  | Time N2 (mins) | 41.26 | 15.74 | 20.00 | 63.50 |
|  | Time N3 (mins) | 18.24 | 12.50 | 0.00 | 40.00 |
|  | Time N2/3 (mins) | 59.50 | 18.01 | 23.00 | 91.50 |
|  | Time REM (mins) | 5.82 | 9.98 | 0.00 | 29.00 |
|  | Total Sleep Time (mins) | 77.71 | 12.24 | 49.50 | 99.00 |
|  | Proportion N1 | 0.17 | 0.15 | 0.03 | 0.66 |
|  | Proportion N2 | 0.52 | 0.15 | 0.28 | 0.78 |
|  | Proportion N3 | 0.24 | 0.16 | 0.00 | 0.55 |
|  | Proportion N2/3 | 0.76 | 0.16 | 0.34 | 0.97 |
|  | Proportion REM | 0.07 | 0.12 | 0.00 | 0.35 |

**Supplementary Table 1.** *Summary sleep statistics.*

| **Metric** | **Session** | **Contact** | **Mean** | **SD** | **Min** | **Max** |
| --- | --- | --- | --- | --- | --- | --- |
| Spindle Rate | Both | C3 | 2.76 | 0.40 | 2.15 | 3.92 |
|  |  | C4 | 2.69 | 0.31 | 2.16 | 3.40 |
|  |  | Cz | 3.16 | 0.53 | 1.96 | 4.24 |
|  |  | All | 2.69 | 0.54 | 0.29 | 4.53 |
|  | 1 | C3 | 2.77 | 0.39 | 2.25 | 3.92 |
|  |  | C4 | 2.67 | 0.25 | 2.28 | 3.14 |
|  |  | Cz | 3.19 | 0.56 | 1.96 | 4.05 |
|  |  | All | 2.71 | 0.55 | 0.29 | 4.35 |
|  | 2 | C3 | 2.76 | 0.41 | 2.15 | 3.81 |
|  |  | C4 | 2.71 | 0.37 | 2.16 | 3.40 |
|  |  | Cz | 3.13 | 0.51 | 2.17 | 4.24 |
|  |  | All | 2.67 | 0.54 | 0.90 | 4.53 |
| Number of Events | Both | C3 | 165.82 | 45.81 | 67 | 268 |
|  |  | C4 | 162.00 | 44.54 | 62 | 258 |
|  |  | Cz | 191.61 | 61.35 | 58 | 350 |
|  |  | All | 161.21 | 49.99 | 24 | 355 |
|  | 1 | C3 | 168.21 | 38.42 | 102 | 258 |
|  |  | C4 | 164.68 | 45.00 | 74 | 258 |
|  |  | Cz | 195.37 | 58.10 | 95 | 350 |
|  |  | All | 164.36 | 45.40 | 24 | 355 |
|  | 2 | C3 | 163.42 | 53.15 | 67 | 268 |
|  |  | C4 | 159.32 | 45.15 | 62 | 240 |
|  |  | Cz | 187.84 | 65.81 | 58 | 328 |
|  |  | All | 158.06 | 54.03 | 49 | 343 |

**Supplementary Table 2.** *Summary spindle statistics for C3, C4, and Cz.*
